# Cajal the artist. The origins of a passion

**DOI:** 10.3389/fnana.2026.1949411

**Published:** 2026-09-29

**Authors:** Jairo A. Rozo, Leonardo Palacios-Sánchez, Andrés M. Pérez-Acosta

**Affiliations:** 1 Iván Pavlov Laboratory, Fundación Universitaria Los Libertadores, Bogotá, Colombia; 2 Neuroscience Research Group, School of Medicine and Health Sciences, Universidad del Rosario, Bogotá, Colombia; 3 Observatory of Self-Medication Behavior, Behavioral Sciences Research Group, School of Medicine and Health Sciences, Universidad del Rosario, Bogotá, Colombia

**Keywords:** neuroscience, drawing, literature, photography, Santiago Ramón y Cajal (1852–1934)

## Abstract

Santiago Ramón y Cajal is widely recognized as the father of modern neuroscience. However, his life has many extraordinary facets, and he is even considered a polymath. Among his many activities, his artistic endeavors stand out, and this article focuses on exploring the origins of Cajal’s passion for the arts. The Aragonese scholar cultivated drawing, painting, literature, chess, and, notably, photography with equal intensity and passion. This article shows Cajal’s artistic dimension, exploring the origins and evolution of his passion for drawing, painting, and photography. We demonstrate that his profound visual capacity and the technical skill developed through his art were not unrelated to his scientific work. On the contrary, they became the fundamental support for his ability to innovate and document the delicate structures of the nervous system through beautiful drawings and photographs that allowed him to show humanity the “butterflies of the soul,” the name he gave to the elegant cells he saw in nervous tissue. His inclination for drawing was evident from childhood, and despite serious conflicts with his father, who desperately wanted him to become a doctor, he managed to take painting courses and perfect his technique. His work as a painter, writer, and photographer is presented, leaving the door open for future explorations into the life of a great man who continues to amaze almost a century after his passing.

*An unrepentant worker, he did not usually waste time on useless conversations. He speaks as he writes, clearly, concisely and with great correctness; he is not one-sided like so many wise men, but rather, on the contrary, he possesses extensive culture: a polyglot, philosopher, writer, draftsman, watercolorist, photographer. He belonged to that type of doctor favored by Letamendi, who, in addition to Medicine, knows many other things and knows them well.*

Roca, cited by Ferrer (1989), p. 27

## Introduction

The authors of this article have developed a line of research on Santiago Ramón y Cajal, studying his life and work (Rozo and Rodríguez-Moreno, 2015; Palacios-Sánchez, 2020), the controversy surrounding his Nobel Prize (Rozo et al., 2017; Rozo et al., 2022) his neuron theory and the idea of plasticity (Rozo et al., 2024, and his work as a painter (Palacios-Sánchez et al., 2015). This current work focuses on exploring the origins of Cajal’s artistic passion.

Santiago Ramón y Cajal (1852–1934) is universally recognized as the father of modern neuroscience, a leading figure in Spanish science who deserved the Nobel Prize in Medicine and Physiology in 1906 (Cajal, 1906/1907); Fernández-Santarén, 2008) for his revolutionary contributions to the neuron doctrine. However, the career of this celebrated scientist was marked by a profound and less explored artistic facet, which led him to cultivate with equal intensity and passion drawing, painting (Palacios-Sánchez et al., 2015), fiction (Collado-Vázquez and Carrillo, 2019), chess (Alonso, 2014), and, notably, photography (Ramón y Caja S., 2007; Museo Archivo Histórico de la Sen, n. d.). From his childhood to his later years, these artistic disciplines were much more than mere pastimes; they shaped his personality, sharpened his powers of observation, and constituted an essential part of his intellectual development.

The central purpose of this article is to recover and revalue this artistic dimension of Cajal, exploring the origins and evolution of his passion for drawing, painting, and photography. To this end, we detail the formative influence and the dialectic of the conflict with his father, Justo Ramón Casasús, a surgeon and anatomist. Finally, we demonstrate that the profound visuality and technical skill developed through his art were not foreign to his scientific work but rather became the fundamental support for his ability to innovate and document the delicate structures of the nervous system.

## The early years

Into a humble family, Santiago Ramón y Cajal was born on May 1, 1852, in the small village of Petilla de Aragón, which, although located in Aragon, administratively belonged to the province of Navarre (Cannon, 1981). This village had ninety-eight houses and a population of just over 800. Mail arrived twice a week, and all the access roads were in terrible condition (López-Piñero, 2000).

According to Alonso-Fernández (2007), Cajal experienced what he calls the “experience of orphanhood,” which may correspond to reality or be the imagined product of a child’s mind fueled by the absence of a loving parental figure (the mother’s silent distance and the father’s constant conflict). According to this author, Cajal’s deep personal motivation stemmed from feeling orphaned, which led him to pursue triumphs and successes. Leaving aside this theory by Alonso-Fernández, which has a distinctly psychoanalytic undertone, it does seem that the paternal figure was fundamental in shaping Cajal’s personality, through his dialectical response of rebellion against his father. Don Justo was a terribly authoritarian, harsh, cold, and pseudo-puritanical father, whom Santiago confronted many times. They had a difficult relationship, based on a dialectic of confrontations, but in one way or another, it was this struggle with his father that possibly forged the will and temperament that made the scientist Ramón y Cajal famous.

## The dialectical and conflictive relationship with the father

Don Santiago refers to his father thus: “With his blood he bequeathed me moral qualities, to which I owe everything I am: the religion of the sovereign will, faith in work, the conviction that persevering and diligent effort is capable of shaping and organizing everything from muscle to brain, compensating for deficiencies of nature and taming even the fatalism of character, the most tenacious and recalcitrant phenomenon of life. From him I also acquired the beautiful ambition to be something and the determination to spare no sacrifices to achieve my aspirations, nor ever to deviate from my path for secondary reasons and petty causes” (Cajal, 1923/2006, p. 100).

Santiago started school at age four in the village of Valpalmas, but his true teacher was his father, who, by age six, had taught him to write correctly and possess a basic understanding of geography, arithmetic, and French. Classes were held in a shepherd cave, allowing him to isolate himself and concentrate on his work (Cannon, 1981). Justo Ramón had begun to instill in his son his own drive for self-improvement (López-Piñero, 2000). When his father went to Madrid to complete his medical studies, young Santiago was entrusted with family correspondence and kept him informed of local events and family news.

Santiago was a rebellious child, passionate about the outdoors, nature observer, and a lover of animals, especially birds. Yet, he was also mysterious and withdrawn, displaying shyness and reserve around adults. He would spend entire days running through the woods and ravines, which caused him problems with his father and his rigid routine.

When Santiago was in Ayerbe, he became the leader of a gang of boys with whom he committed various pranks, such as raiding orchards. He learned to handle the club, the arrow, and the sling with remarkable skill and crafted breastplates, helmets, and arrows for the organized wars in which they participated. He acquired great expertise in arrow-making (Cannon, 1981), taking advantage of his friendship with the shoemaker apprentices to obtain the raw material: broken awls. It was precisely during this time that his artistic inclinations began to emerge.

## The “War” for drawing

He then began to draw everything he saw: villagers, carts, cats, dogs, horses, landscapes. The next step was to saturate his drawings with colors, and he began to cultivate this skill to get the most out of the smallest things. Since he had no way to acquire paint, and later his father forbade him from painting, Santiago began to create alternatives to obtain colors. He scraped paint off the walls or soaked the intensely colored lining of cigarette paper booklets. Since he had no brushes, he used rolled-up colored papers, which, when soaked with water, released their color. Due to his artistic tendencies, he began to embrace solitude and isolation to develop his favorite pastime. Both factors–the ability to reproduce images and a predisposition to overcome material difficulties with alternative procedures–would be fundamental in the scientist’s future career (Cannon, 1981; Baratas and Santesmases, 2001).

But Santiago was immersed in an environment that was not conducive to his passion. The sacred fear of poverty that life had instilled in Don Justo Ramón’s heart transformed Cajal’s father into an exaggerated and almost paranoid positivist who despised all literary or artistic culture as unnecessary ornamentation. Don Justo projected all his frustrations and obsessive traumas onto Santiago. The Ramóns’ family life unfolded under an almost tyrannical regime of exacerbated economic foresight. Cajal would later lament such a sad and meager ideal of life. For the Ramóns, there was no art, no music, no reading of novels, no theatrical performances (Cannon, 1981). Don Justo believed that man should acquire and use his knowledge for profitable purposes, and that the child should be educated by the most direct and quickest path to face life’s responsibilities. He considered painting a second-rate profession that clashed with what he had planned for his son. Therefore, Don Justo decided that Santiago should become a doctor, even before he turned ten. However, for young Santiago, his father’s refusal fueled his love for painting. In his work, “My Childhood and Youth,” he writes about his father’s opinion on art: “He promised me that when I became a doctor, that is, at twenty-one, with my financial situation secure, I could wander as much as I wanted through the fantastical realms of art” (Martínez Falero, 2011).

Cajal began to draw landscapes, devastating wars featuring Greek and Roman heroes, as well as active, not contemplative, saints. One of these was a portrait of the Apostle James (patron saint of Spain), whom he painted on horseback, fearless, with a wild beard, sword and shield, standing on a field of Moors. Tired of confiscating papers, brushes, and pencils, his father decided to resolve the problem once and for all. Since there was no one in the village with sufficient knowledge, he resorted to submitting his son’s work to the judgment of a facade plasterer and decorator, a stranger who had come to Ayerbe to repair the church damaged by fire. The verdict was entirely negative. Whereupon, after a family meeting, Don Justo decided that Santiago should abandon art and become a doctor. All the tools that would allow him to cultivate his artistic talent were confiscated, and a war began between Don Justo and Santiago that lasted 12 years and which Don Justo ultimately won, as Santiago became a doctor. However, his special predilection and aptitude for drawing ended up shaping his scientific and professional career (Ramón y Cajal M., 2007).

This “war over drawing” had several important battles. Don Justo decided to send Santiago, at the age of ten, to study in Jaca (in 1861), to attend the Piarist school while living with a maternal uncle. The school was ruled by an iron discipline and Father Jacinto’s “reign of terror,” which included punishments such as fasting, confinement, beatings, and humiliation, all suffered by Santiago for his constant rebellion. On the verge of expulsion, he only passed the course because the examining board was made up of professors from the Huesca Institute, and one of them was a friend of Don Justo (Cannon, 1981).

When Santiago returned to Ayerbe, his condition was so lamentable that his mother was frightened to see him. Don Justo also found the school’s strictness excessive and transferred him to the Institute in Huesca, where he completed the rest of his secondary education. From that institute, Don Santiago fondly remembered his Latin and geography teachers, and the subjects that positively influenced him were rhetoric and poetics, physics, and chemistry with their fabulous laboratory experiments. On the other hand, he had no interest in history, psychology, logic and ethics, or Greek. Cajal, as Cannon (1981) states, was a student of inconsistent dedication and poor performance. To try to guide him, his father decided that he should combine his studies with working as a barber’s apprentice, and when he failed Greek, he interrupted his studies for a year, placing him as a shoemaker’s apprentice in Gurrea de Gállego, one of the small villages where Don Justo worked. Throughout this time, Cajal took refuge in drawing, surrendering to his passion while enduring the grueling school year in Jaca, while a student in Huesca, while apprenticed as a barber or shoemaker, and utterly dissatisfied with the world he lived in, he retreated into himself, as he says in his “Memoirs” (Cajal, 1923/2006).

When his father ordered him to return to the Institute in Huesca, Santiago promised to study seriously on the condition that he enrolled in a drawing course offered at an academy in Huesca, and Don Justo had no choice but to accept. Santiago won this battle and earned the first top mark of his life (López-Piñero, 2000). As his granddaughter reminds us, after the humiliating verdict of the revocation committee, the knowledgeable teacher at the Institute, León Abadías de Santolaria, awarded him a distinction and a prize, and even advised his father to send him to study Fine Arts, because there was more to his artistic inclinations than a mere passing hobby. But it was all in vain (Ramón y Cajal M., 2007). Don Justo refused to budge; Cajal would become a doctor. Don Justo’s demands tempered Santiago’s personality like steel, and the more problems he faced in life, the harder he worked to find solutions.

During this period when he painted everything, Santiago began to work on his appreciation of chromatic tones and decided to create a kind of color dictionary in which he set out to reproduce all the nuances of natural objects, assigning each color a numerical value. While this systematization involved rocks, insects, and wildflowers, Santiago achieved his goals. However, when it came to cultivated flowers (he began to run into trouble with their owners) and other objects, the difficulty increased (Leirós, 1977). Interestingly, in this kind of systematic fever with color, his methodical and orderly spirit began to take shape, a spirit that would be fundamental to his later scientific work.

## Cajal and literature

Cajal’s artistic expressions were related to drawing, but also to literature and photography. Don Justo considered novels and literature to be no more highly regarded than painting; therefore, Santiago could only read a few novels of little literary merit in his childhood, which his mother kept in a trunk from her single days, and, of course, secretly from Don Justo (López-Piñero, 2000). But in the summer of 1863, Santiago discovered a complete library in the attic of a confectioner neighbor. Secretly and without anyone’s permission, he took them out one by one and read them avidly. Once he had read them, he put them back in their place, and in this way, he became acquainted with Dumas, Quevedo, Cervantes, Victor Hugo, and others, immersing himself in the world of Romantic literature (Cannon, 1981).

This love of literature remained with him throughout his life (until the year of his death: Cajal, 1934/1939, see Figure 1), and he even ventured into fiction, writing and publishing several short stories as *Holidays Stories/Pseudoscientific narratives* (Cajal, 1906, see Figure 2). Cajal had also begun other books of a philosophical and literary nature, such as *The Mystery of the Tomb* and *Hallucinations and Dreams*, or *Notes on Histological Psychology, Hypnotism, and Suggestion.* These manuscripts were lost during the Spanish Civil War (Cannon, 1981).

**FIGURE 1:**
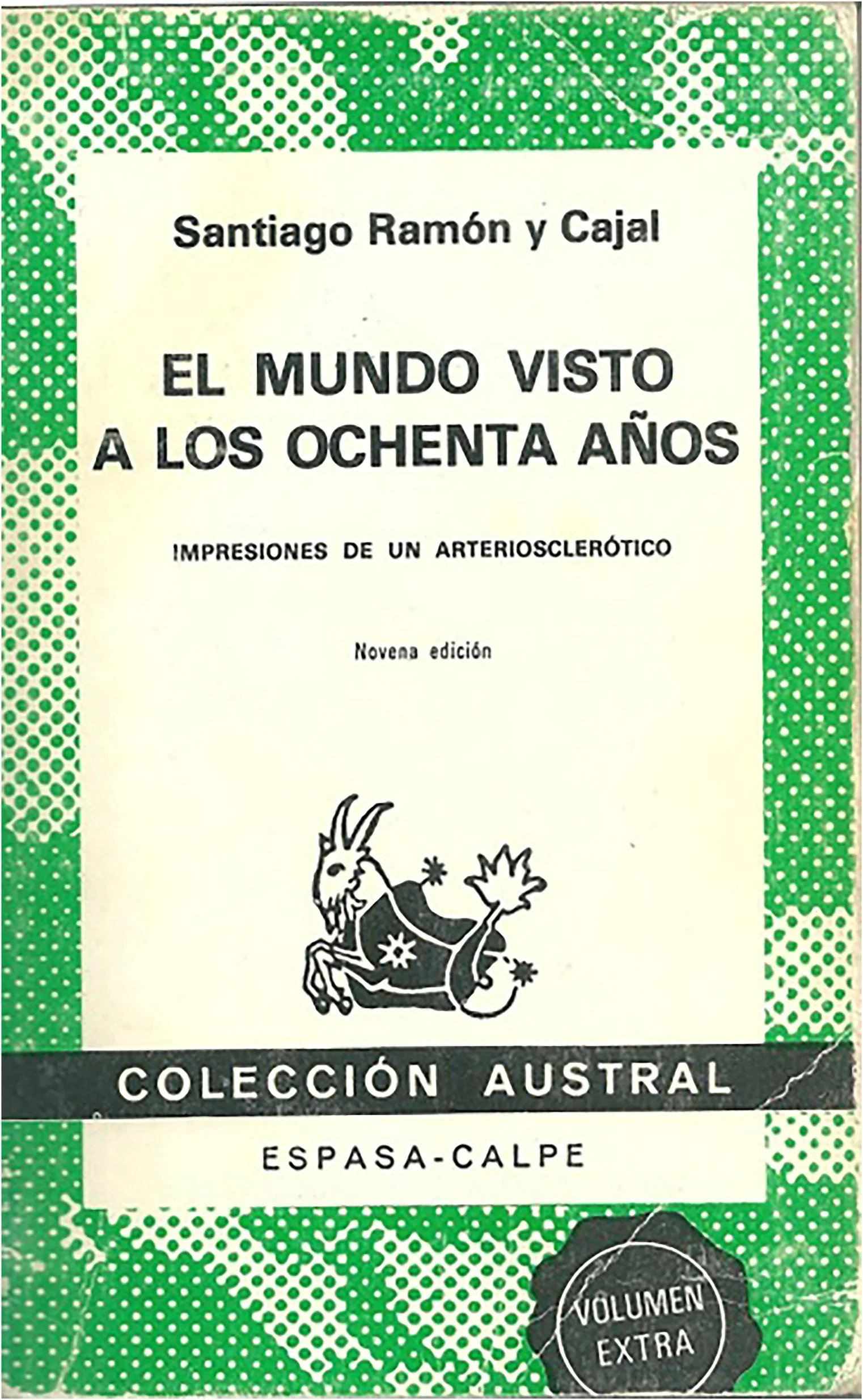
Cover of the book “The World Seen at Eighty Years Old.” Own source. Property of Jairo A. Rozo.

**FIGURE 2:**
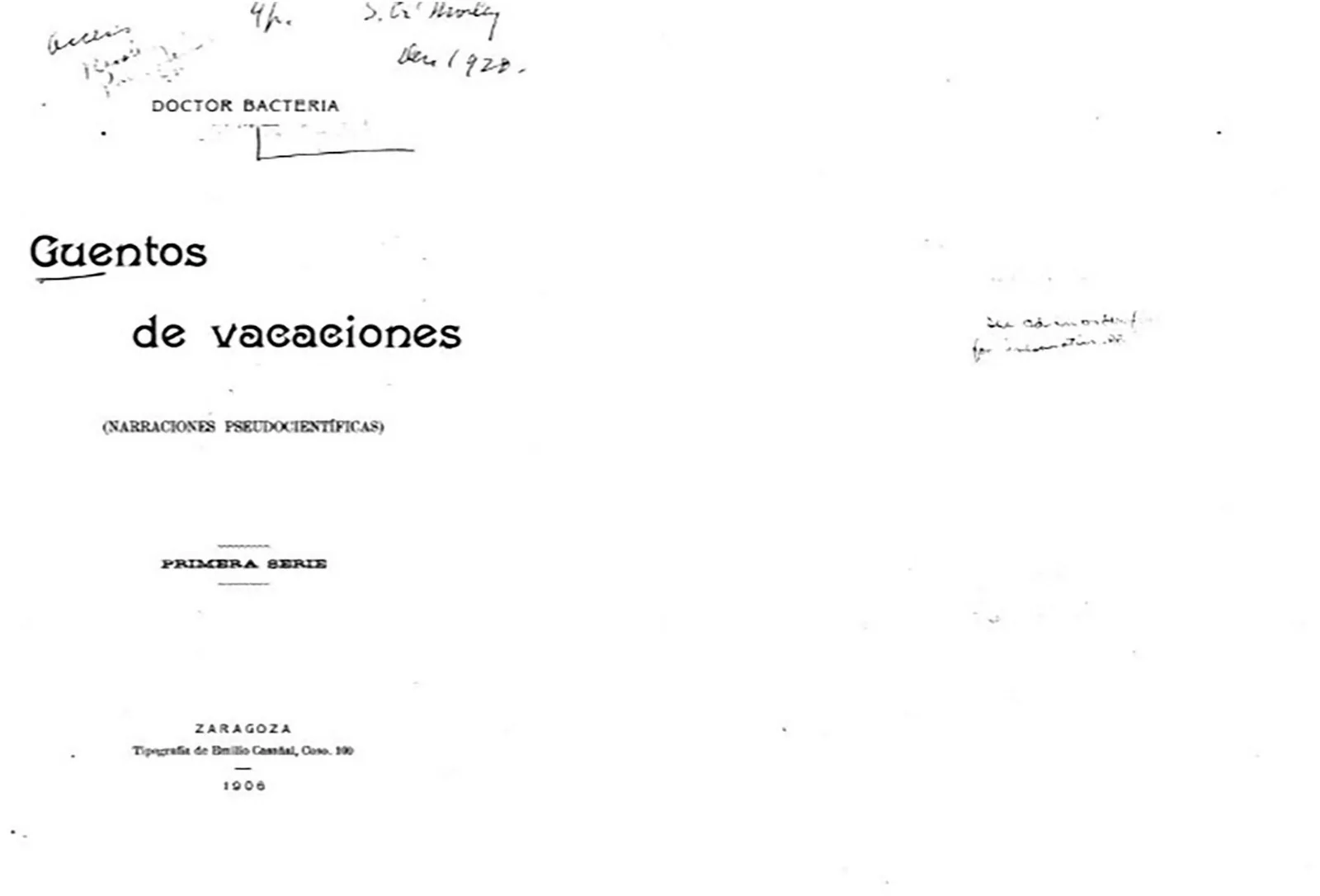
Cover of the book “Holiday Stories. Pseudoscientific Narratives.” Image in the public domain. Source: https://archive.org/details/cuentosde vacaciones1906/page/n7/mode/2up.

## Cajal and photography

One of the Cajal greatest passions was photography (including his personal and familiar portraits and self-portraits: see Figures 3, 4). Cajal’s first encounter with photography was the discovery of its physical principle: the image reflected in a darkroom. This occurred by chance, while he was serving a school punishment locked in a basement. There, he had the good fortune to discover the physical phenomenon that Leonardo da Vinci and Giacomo della Porta studied their perspective reproductions (Romero, 1984). While still in Ayerbe, he was punished at school for his famous caricatures, which were passed around among the students. He was locked in the dungeon, a very dark basement infested with mice, where the dim light filtered through the cracks of the small window overlooking the town square. Not knowing what to do, Santiago stared at the ceiling and noticed that the ray of light entering the room projected everything passing by in the square upside down. With the help of paper and saliva, he narrowed the gap in the window and discovered, to his delight, that the figures had become clearer. It was a great discovery for him (Leirós, 1977).

**FIGURE 3:**
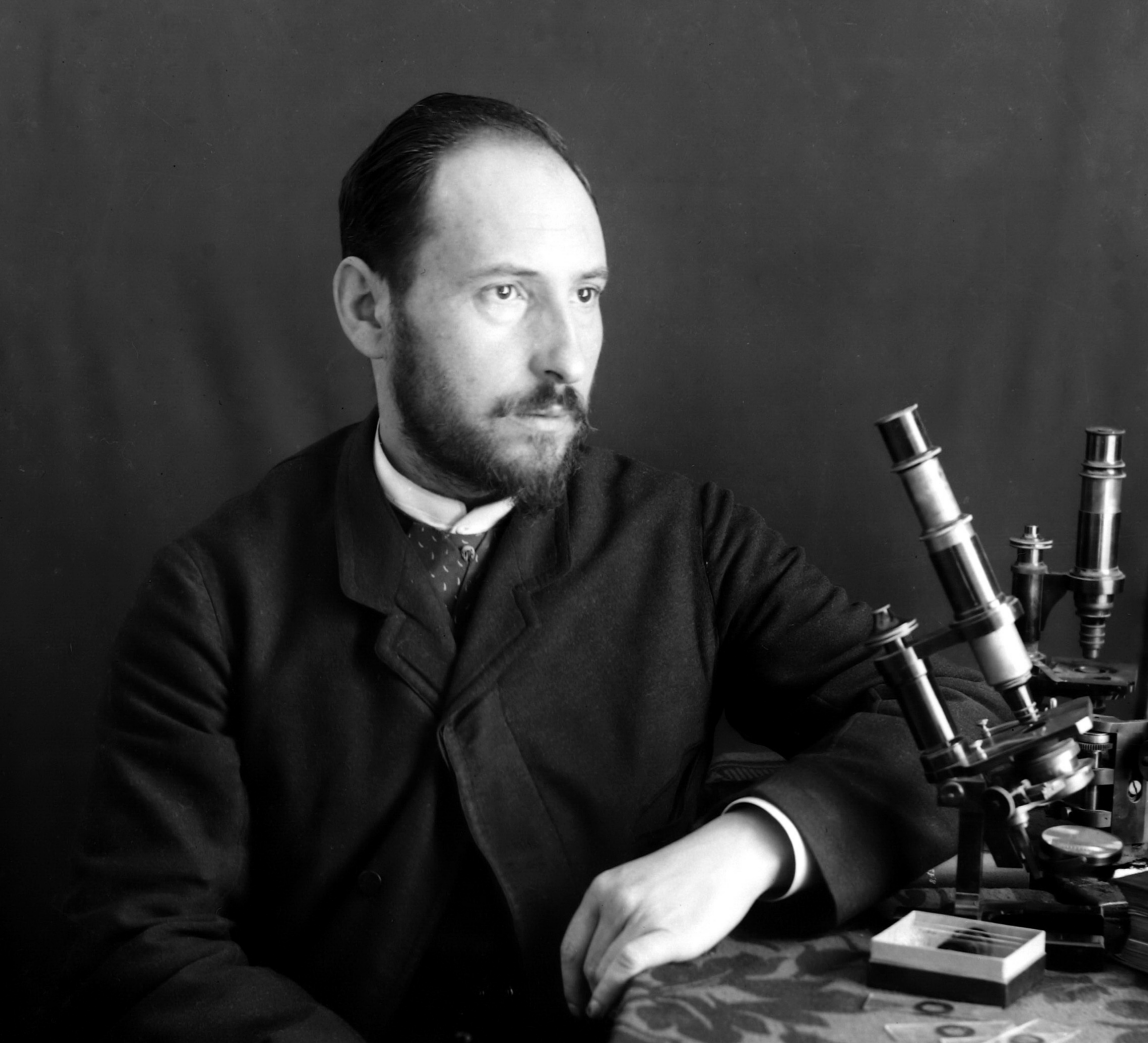
Santiago Ramón y Cajal (1 May 1852 – 18 October 1934) portrayed with a Carl Zeiss microscope (image adapted with permission from ‘Santiago Ramón y Cajal’, https://commons.wikimedia.org/wiki/File:Santiago_Ram%C3%B3n_y_Cajal_(8691434605).jpg, licensed under CC BY-SA 2.0).

**FIGURE 4:**
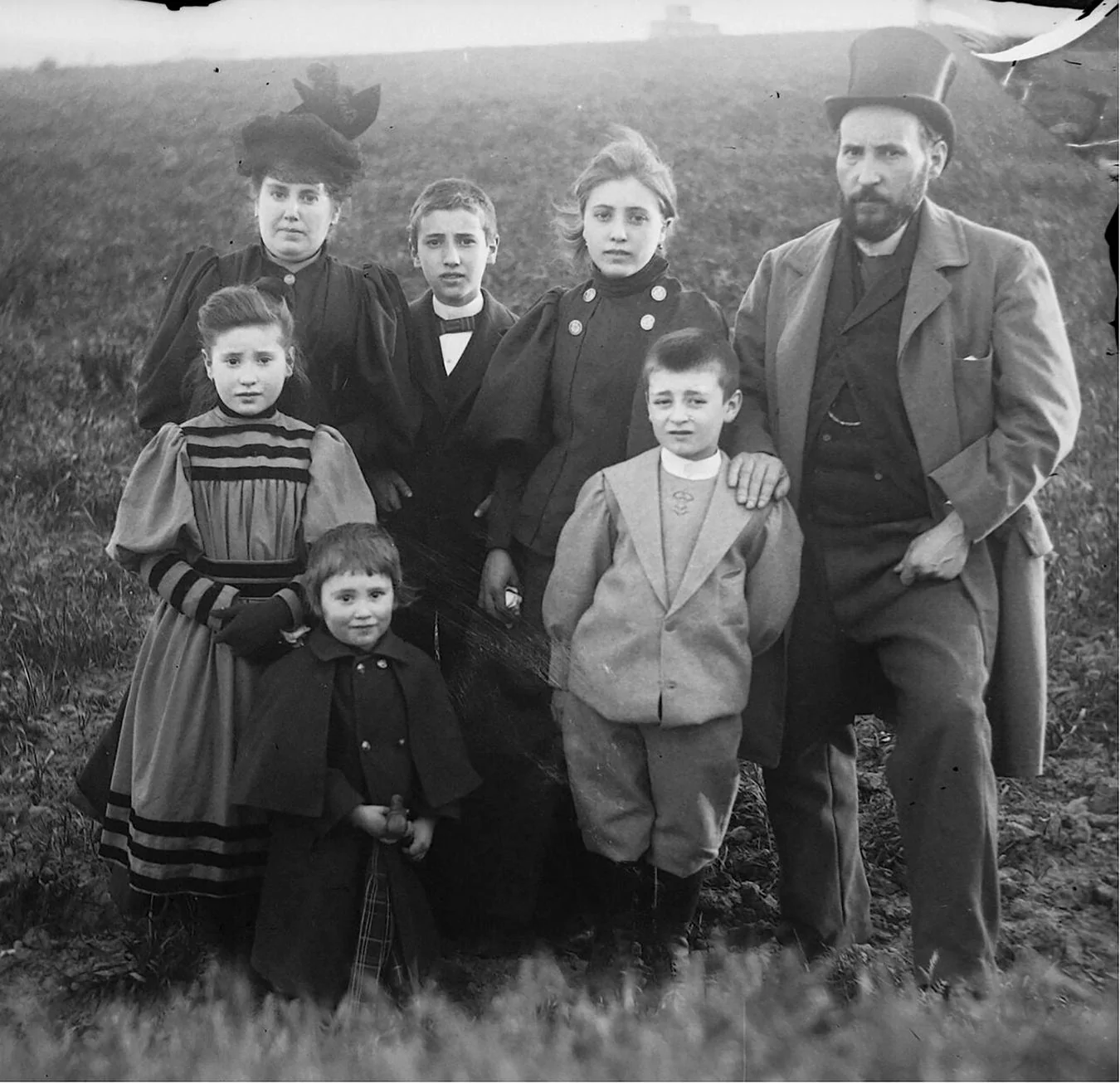
Family self-portrait of Cajal with his wife, Silveria, and his children, *circa* 1895. Image in the public domain. Source: https://es.wikipedia.org/wiki/Archivo:Autorretrato_familiar_de_Cajal_con_su_esposa,_Silveria,_y_sus_hijos,_hacia_1895.jpg.

But the true impact photography had on him occurred in 1868 in the city of Huesca. Cajal was already familiar with photography and had encountered itinerant photographers practicing Daguerre’s primitive method (Ramón y Cajal M., 2007). Photography, or the daguerreotype, had been introduced to Spain by the physician Pedro Felipe Monlau in 1839 (López-Piñero, 2000), but what astonished Cajal was learning firsthand about the wet collodion process, a difficult procedure that requires a heroic struggle with the unpredictable silver plating and the agonizingly slow exposure time. Thanks to a friend who knew some photographers, he was able to enter the darkroom, learn their techniques and the materials they used, including, in addition to wet collodion, silver iodide, silver bromide, pyrogallic acid, and the sensitization of albumen paper (Ramón y Cajal M., 2007; Romero, 1984).

From the age of 18, he developed a passion for photography, learning all the tricks of the trade. For 50 years he nurtured his passion and witnessed all the evolutionary stages of photography as the years passed, from the daguerreotype plate, the wet collodion process, and the gelatin-bromide processes of Bennett and Monckhoven, where minutes became fractions of a second and where it was possible to capture the snapshot of movement. In his later years, he mastered Vögel’s auto chromatic process and silver emulsions, eventually arriving at new techniques for photographing colors (Romero, 1984). “Photography was one of Cajal’s great passions. Before becoming a neuroscientist, Cajal was a chemist, and thanks to his knowledge, mostly the result of self-study, he was able to master the photographic techniques of the time” (Espeleta and de Carlos, 2023, p. 22).

Drawing, photography, and literature were three passions that, over time, became invaluable allies for his scientific work, where they converged, offering him a different vision that made him superior to the scientists of his time.

## Art and science converge

In the autumn of 1868, Cajal began to take his studies seriously. Subjects such as physics, chemistry, and natural history began to hold a special interest in Santiago. He was fascinated by chemistry and physics experiments and began to study mathematics on his own, discovering that the entire universe, from the infinitesimally small to the infinitely large, was constructed according to formulas (Cannon, 1981; Ramón y Cajal M., 2007). Seeing this new attitude from Santiago, Don Justo finally began to hope that he would become a good doctor and therefore enrolled him at the University of Zaragoza. In September 1869, Cajal enrolled in the preparatory course, the first of those that made up the medical education plan in effect at that time (López-Piñero, 2000).

In 1869, Justo Ramón was determined to make his son a doctor—as Cajal himself stated (1923/2006)–and they traveled to Zaragoza to enroll in the preparatory year courses. Cajal was 17 years old and entered the Zaragoza School of Medicine. To prevent young Santiago from returning to his old ways, he arranged for him to work as an apprentice in the home of a friend, fellow countryman, and colleague, Mariano Bailo, who had an excellent reputation as a surgeon. Fortunately, in 1870, Justo passed the competitive examination for Physician of the Provincial Charity and obtained a position, so he moved to Zaragoza with his family. This gave him the opportunity to personally supervise Santiago’s studies. Furthermore, shortly afterward, he was appointed Acting Professor of Dissection at the School where his son was studying. He immediately dedicated himself with zeal and fervor to turning young Santiago into a skilled dissector (Cannon, 1981).

Father and son began studying anatomy, dissecting cadavers in the dissection room of the Hospital de Santa Engracia, and worked diligently, shoulder to shoulder, for 3 years, examining each part–muscles, glands, nerves–and meticulously verifying what was written in the anatomy books. In the end, Cajal saw in the cadaver not death, but the admirable artifice of life (Cajal, 1923/2006; Cannon, 1981).

As reference books, the Cajal’s had Jaime Bonells and Ignacio Lacaba’s Complete Course of Human Anatomy, likely an 1820 edition, or the four volumes of M. Philibert Sappey’s *Traité d’anatomie descriptive*, a Spanish edition from 1854 to 1858. During this time, a new relationship developed between father and son. After the internal struggle they had experienced, irritation and severity had now been replaced by paternal indulgence (López-Piñero, 2000). Such was the change between the two that drawing became a source of pride for Don Justo, as Santiago faithfully and beautifully depicted the intricate structures of anatomical specimens. In fact, Justo considered publishing an Atlas of Anatomy, beautifully illustrated with Santiago’s drawings, but this project was never realized due to the economic and technical limitations of the time, as color reproduction and graphic arts were quite underdeveloped in Spain (Cannon, 1981).

Cajal revived his interest in photography to adequately illustrate his early publications. In 1880, he published his first article on inflammation of the mesentery, cornea, and cartilage, and in 1881, he published his second article on nerve endings in voluntary muscles. These were pamphlets of fifty and sixty pages, illustrated with two lithographic plates, of which he printed 100 copies at his own expense (López-Piñero, 2000). These works, published in Zaragoza and distributed among his friends, naturally did not achieve any widespread dissemination or recognition (Ramón y Caja S., 2007).

Cajal made significant progress in the fields of photography and drawing, learning to produce “ultrafast gelatin-bromide photographic plates,” a technique uncommon in Spain at the time. He made them at night with the help of Doña Silveria in a makeshift workshop in the barn of their house. These plates proved so successful that he earned extra income by selling them to professional photographers. Eventually, he learned lithographic techniques and applied photography to the reproduction of these types of illustrations (López-Piñero, 2000).

Returning to his drawings, his representations of nervous tissue are scientific expressions, but they reveal the great pictorial and artistic skill of the father of modern neuroscience. In 1888, using the Golgi method, he depicted dendritic spines in his drawings for the first time, demonstrating his remarkable ability to interpret histological preparations by drawing various elements that many other scientists overlooked (Osorio, 1984). He managed to combine his artistic talents with the ability to decipher histological preparations, and as a result, he obtained accurate and precise drawings of the neuronal organization and structure of the nervous system. Therefore, drawing was not a simple task, since the scientist had to discard what he considered artifacts and draw what he believed to be part of the tissue, thus avoiding the possibility of misinterpretations (Palacios-Sánchez et al., 2015). His drawings are comparable to those of Leonardo da Vinci or Michelangelo Buonarroti, faithful copies that show the micro-organization of the nervous system (Espeleta and de Carlos, 2023).

Regarding his skills, the combination of his innate abilities and those acquired under the tutelage of León Abadías allowed him to develop an ideal technique that reflected great precision, dexterity, and delicacy in his numerous scientific drawings of the structure of the human body and, specifically, the nervous system. In creating his drawings, he used two types of paper: the first was writing paper, used for his small-format works (between 21 cm × 15 to 30 cm); and the second was kraft paper, used for his mural-sized works, which measured more than 1 meter high by more than 80 cm wide (Meseguer, 2014).

Most of his small-format drawings were done freehand (see De Felipe, 2006): with pencil, charcoal, pen, or brush, later adding black India ink and, occasionally, other colors to achieve an effect even closer to the histological slides he observed. For his mural-sized drawings, he used charcoal to create an initial sketch of the image and subsequently added colored chalks, which served as a tool to provide intensity and support to the background of the image. To enhance the forms, he implemented dark patches, shading, and lines that emphasized the contours of the structures, on a background impregnated with washes (white and grayish watercolors) that brought light and color to the drawings. Additionally, small, consecutive lines and fine pencil stippling gave the images a voluminous effect. He managed to provide his illustrations with a sense of depth, which gave the drawing an appropriate scale; it demonstrated the undeniable artistic vocation that accompanied him throughout his scientific career (Meseguer, 2014).

## Technique, art, science, and neuroaesthetics

### Technical limitations and the role of illustration in early histology

In the late 19th and early 20th centuries, technological limitations—such as the lack of photomicrographs and the pronounced distortion at the edges of optical lenses—necessitated representing observations of the nervous system through drawings. For this reason, the researcher’s judgment and interpretation played a crucial role in the creation of the final graphic representation (Weller, 2011). In the past, neurohistological research required artistic skills to disseminate observations. However, instead of meticulously replicating the sample, researchers visually selected elements relevant to their hypothesis. This subjective filter introduced biases that could hinder scientific consensus. Despite this, a rigorous drawing maintains its documentary relevance permanently, as it transcends the theoretical readings of its time. As Cajal emphasized at the beginning of the 20th century, the attraction to the study of the brain stems from an aesthetic sensibility capable of appreciating and capturing the brain’s microarchitecture, inaccessible and incomprehensible to the average observer (De Felipe, 2010).

### Scientific rigor, synthesis, and empirical validation in Cajal’s work

During Cajal’s time, hand-drawn illustrations were the primary means of disseminating microscopic observations, leading the scientific community to react with some skepticism and interpret them as stylized compositions rather than objective replicas. However, Cajal’s illustrations transcend their aesthetic merit: they are extremely rigorous representations that map the organization, pathways, and connectivity of neurons. His greatest contribution lay in his ability to synthesize the architecture of nervous tissue, combining technical skill and analytical interpretation to discern the actual structural details from the prepared specimens. The accuracy of his work has been subsequently confirmed through the re-examination of his histological samples and the use of contemporary technologies (De Felipe and Jones, 1988, 1991). Therefore, although, as Fernando de Castro pointed out, his drawings elevated science to an artistic level, their primary value lies in having laid the empirical foundations of modern neuroscience (De Felipe, 2006).

### Visual language, pictorial techniques, and communication strategy

Cajal’s significance stems not only from his scientific discoveries but also from the consolidation of a unique graphic language and an effective dissemination strategy that combined traditional drawing and photography. Cajal combined scientific objectivity with a personal narrative about the brain and his own role, using photographic self-portraits to make his figure visible and amplify the impact of his histological diagrams. Unlike the current conception of scientific illustration as mere empirical data, nineteenth-century figures functioned as argumentative resources. Cajal constructed his own expressive system by integrating microscopic observations, staining techniques, hand-drawn illustrations, and photography. His images employed three-dimensional optical resources and Renaissance painting techniques–such as chiaroscuro and sfumato, learned from his teacher León Abadías–to prioritize information and convey ideas that words could not encompass. Thus, his artistic training was not an incidental trait, but rather the cornerstone of his observational rigor and the enduring cultural significance of his legacy (Hunter et al., 2025).

Santiago Ramón y Cajal’s success stemmed not only from his biological discoveries but also from the development of his own unique visual language and an innovative personal branding strategy (f. i. the beautiful image of Purkinje cells in Figure 5). Cajal intertwined scientific objectivity with a subjective narrative, drawing on intuition to transcend empirical limitations and suggest that the complexity of neural connections underpinned intelligence (see De Felipe, 2006). Since microscopic samples often present severed or incomplete structures, he created conceptual reconstructions by combining elements from multiple preparations and filling in observational gaps with well-founded assumptions (see, for example, the case of hippocampus structure and functioning with the help of arrows in Figure 6). This method of interactive layering anticipated principles of visual communication such as the multiplane camera, making perspective the graphic pillar with which he translated the spatial relationships of the microscopic world (Hunter et al., 2025).

**FIGURE 5:**
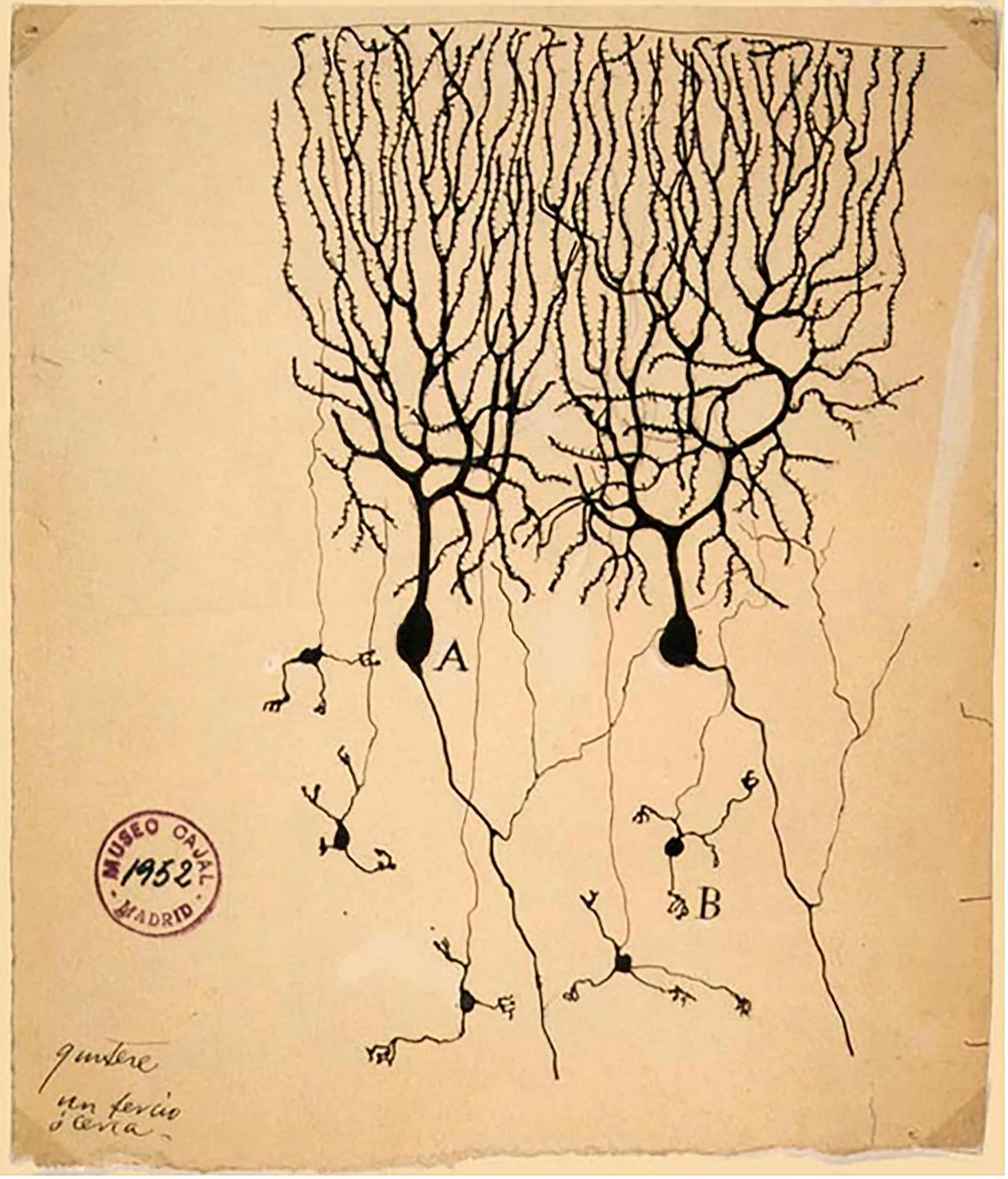
Drawing of Purkinje cells **(A)** and granule cells **(B)** from pigeon cerebellum by Santiago Ramón y Cajal, 1899; Instituto Cajal, Madrid, Spain. Image in the public domain. Source: https://commons.wikimedia.org/wiki/File:PurkinjeCell.jpg.

**FIGURE 6:**
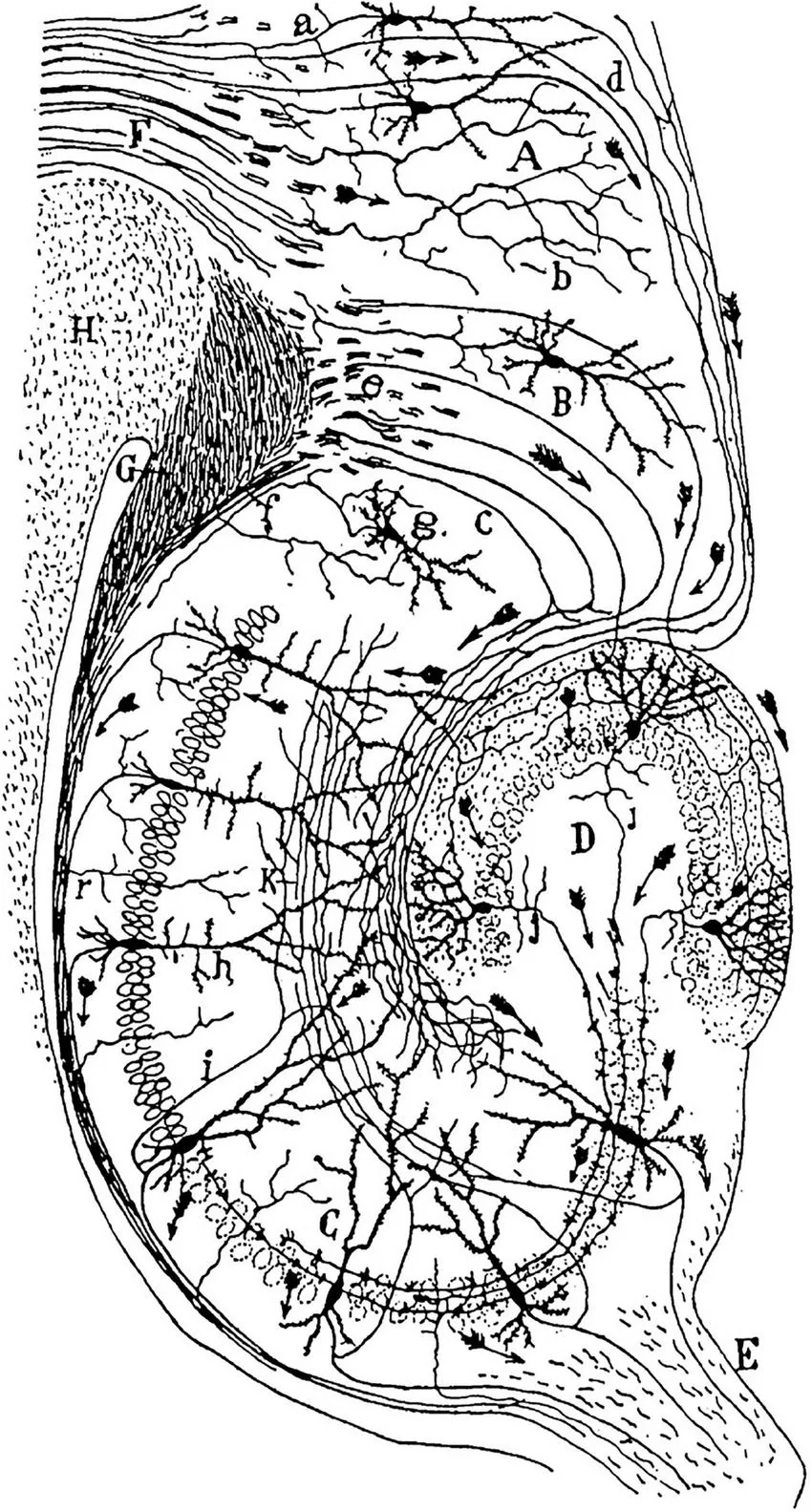
Drawing of the neural circuitry of the rodent hippocampus. By Santiago Ramón y Cajal. *Histologie du Systeme Nerveux de l’Homme et des Vertebrés*, Vols. 1 and 2. A. Maloine. Paris. 1911. Image in the public domain. Source: https://commons.wikimedia.org/wiki/File:CajalHippocampus.jpeg.

### The perspective of neuroaesthetics and abductive reasoning

This approach allows us to draw on neuroaesthetics to examine the role of art in scientific innovation. Through the graphic work of Santiago Ramón y Cajal, it is demonstrated that the aesthetic dimension grants the epistemological resources necessary to challenge prevailing paradigms demonstrate that scientific progress sometimes requires an artist’s perspective. Artistic practice not only facilitates the interpretation of technical images but also reconfigures the discipline’s conceptual frameworks. In Cajal’s case, drawing functioned as a heuristic and interpretive tool that allowed him to infer neuronal connections or synapses, activating cognitive, sensorimotor, and emotional dimensions that revealed the dynamics of the nervous system (Poole, 2022).

Comparing Cajal with Camillo Golgi, it is observed that, although both shared the same technical methodology (staining and the microscope), their rhetorical frameworks and approaches to drawing differed. While Golgi used sketching as a mere functional record conditioned by his theoretical expectations, Cajal employed pictorial practice to integrate multiple perspectives, scales, and temporalities (see De Felipe, 2006). This aesthetic discipline acted as a mechanism of abductive reasoning, allowing him to isolate the individual neuron long before the advent of electron microscopy. Thus, neuroaesthetics confirms that the aesthetic experience transforms conceptual understanding, turning the act of drawing into a reflective exercise capable of deciphering the complexity of the brain (Poole, 2022).

## Conclusion and final comments

The career of Santiago Ramón y Cajal demonstrates that his scientific success was built upon an artistic vocation developed from an early age. This passion took root in his childhood through self-teaching and rebellion against a father who forbade and belittled such expressions and who only came to accept and value them when, as an adult, this artistic ability manifested itself in the copying of anatomical figures under his strict guidance. Although the young Cajal was drawn to the brush and palette, due to his father’s influence he trained as a physician and scientist, but his initial inclinations laid the foundation for the development of visual precision and manual dexterity that would be crucial skills in his later career as a neurohistologist. Let us recall that Cajal himself said that thanks to his childhood artistic pursuits (to which his father always opposed) he owed what he was as a scientist, thanks to the creation of thousands of drawings of the nervous system that allowed him to support his neuron theory (De Felipe, 2025).

His artistic sensibility and profound capacity for observation manifested themselves in two main areas: drawing and photography. Drawing was not only a form of expression and an exercise in visual memory, but it also became an indispensable tool for faithfully documenting his microscopic findings, leading to the development of his precise “Cajal method.” At the same time, photography, to which he dedicated himself fervently from 1870 onward, became both a personal passion and a scientific method. His role as a pioneer of photomicrography in Spain and founder of the Spanish Photographic Society (Pariente, 2024; Ramón-Alonso, 2024) testifies to how he integrated the art of light with his scientific pursuits.

Ultimately, Cajal’s career and legacy can only be fully understood considering his artistic/scientific duality. The Nobel laureate himself acknowledged that his success was largely due to his training and manual dexterity, skills cultivated with perseverance in his artistic activities (Cajal, 1923/2006; Grant, 2007; Jones, 2011). This work recovers and revalues this forgotten dimension, concluding that the artist’s meticulous observation, combined with the scientist’s rigorous methodology, proved to be the unique combination that allowed him to unravel the mysteries of the brain and establish himself as one of the most important figures in universal science.
